# Pregnancy Outcomes Among Women with Treated Iron Deficiency Anemia: A Retrospective Cohort Study

**DOI:** 10.3390/nu17193168

**Published:** 2025-10-08

**Authors:** Threebhorn Kamlungkuea, Chutima Kaewchung, Netjantra Sublon, Nuchpawee Tanyongmasakul, Surangfahom Butsart, Passkorn Winijchai, Phudit Jatavan, Theera Tongsong

**Affiliations:** 1 Department of Obstetrics and Gynecology, Faculty of Medicine, Chiang Mai University, Chiang Mai 50200, Thailand; 2 Fetal Center, Faculty of Medicine, Chiang Mai University, Chiang Mai 50200, Thailand; 3 Faculty of Medicine, Chiang Mai University, Chiang Mai 50200, Thailand

**Keywords:** anemia, fetal growth restriction, iron deficiency, low birth weight, pregnancy outcomes, preterm birth

## Abstract

Background and Objectives: Iron deficiency anemia (IDA) is the most common cause of anemia in pregnant women and can adversely affect both maternal and fetal health. This study aimed to compare pregnancy outcomes between women with and without IDA in Northern Thailand, a region with a high prevalence of anemia. Methods: A retrospective cohort study was conducted on all singleton pregnancies attending antenatal care (ANC) and/or delivering at Maharaj Nakorn Chiang Mai Hospital between 2003 and 2024. The study group consisted of women diagnosed with IDA in the first half of pregnancy, while the control group comprised women with low-risk pregnancies during the same study period. Results: Of the 38,979 pregnancies, after applying exclusion criteria, 634 pregnancies (2.2%) with laboratory-confirmed IDA and 28,132 controls remained available for analysis. Women with IDA had significantly higher parity, lower socioeconomic status, and lower hemoglobin levels throughout pregnancy. Multivariate regression analysis revealed that IDA was significantly associated with increased risks of preterm birth (adjusted odds ratio; aOR 1.04; 95% CI: 1.01–1.07), fetal growth restriction (FGR) (aOR 1.02; 95% CI: 1.00–1.04), and low birth weight (aOR 1.05; 95% CI: 1.03–1.08). Conclusions: IDA, even with treatment, may still slightly increase the risk of adverse pregnancy outcomes, particularly preterm birth, fetal growth restriction, and low birth weight. The residual risk likely reflects incomplete correction of anemia. Optimizing management requires strict compliance, judicious use of parenteral iron, and attention to coexisting nutritional deficiencies, underscoring the need for closer monitoring and improved care strategies.

## 1. Introduction

Anemia is the most common hematological disorder in pregnancy, affecting approximately 36.5% of pregnancies worldwide [1], with a higher prevalence of 35–75% in developing countries [1,2,3]. It contributes to up to 20% of maternal deaths, particularly in Africa and South Asia. In Thailand, the prevalence of anemia among pregnant women ranges from 4.6% to 40% and has shown a steady increase over recent years [4,5].

Iron deficiency anemia (IDA) accounts for nearly 80% of anemia in pregnancy [6], affecting up to half of all pregnant women globally [7,8,9]. IDA is associated with maternal complications, including postpartum hemorrhage and blood transfusion, and adverse fetal outcomes such as preterm birth and low birth weight. Iron is essential for maternal hematopoiesis, placental growth, and fetal development throughout gestation [10,11]. Fortunately, IDA can be diagnosed through laboratory testing and effectively managed during pregnancy, reducing the risk of complications [8,12].

Although the adverse effects of IDA are well recognized, it remains uncertain whether treated women achieve outcomes comparable to non-anemic women. Based on a Cochrane systematic review [13], despite the high incidence and burden of IDA in pregnancy, further research on the treatment of IDA in pregnancy is strongly needed. We hypothesized that early detection and treatment of IDA could lead to pregnancy outcomes similar to those in women without anemia. This study therefore aimed to evaluate pregnancy outcomes among women with IDA diagnosed in early gestation who received adequate treatment and standard antenatal care. The primary objective was to compare the prevalence of fetal growth restriction (FGR) between women with treated IDA and normal low-risk pregnant women, and the secondary objective was to compare other adverse outcomes, including preterm birth, low birth weight, preeclampsia, and postpartum hemorrhage.

It should be noted that this study did not aim to directly determine the natural impact of untreated IDA, which is both ethically unacceptable and confounded by numerous factors. Instead, our objective was to evaluate the extent of obstetric complications among women diagnosed with IDA who received treatment and standard antenatal care, with the expectation that the findings could contribute to improvements in maternal and fetal care.

## 2. Patients and Methods

This retrospective cohort study was conducted on patients who attended the antenatal care clinic and delivered at Maharaj Nakorn Chiang Mai Hospital, a tertiary university teaching hospital. Data were obtained from the obstetric database of the Maternal-Fetal Medicine Unit, Department of Obstetrics and Gynecology, Faculty of Medicine, Chiang Mai University, between 2003 and 2024, supplemented by complete medical records. The study was approved by the Institutional Review Board (Committee 5; Study Code: OBG-2568-0191; Research ID: 0191; approval date: 2 May 2024).

The study population consisted of two groups: (1) women with iron deficiency anemia (IDA), who received standard antenatal care along with IDA treatment (oral ferrous fumarate 200 mg or elemental iron 67 mg three times daily), and (2) non-anemic, low-risk pregnant women serving as controls, who received standard antenatal care with routine iron supplementation (oral ferrous fumarate 200 mg once daily). The inclusion criteria for the study group were: (1) singleton pregnancy; (2) known gestational age based on clinical estimation and first-half pregnancy ultrasound biometry; (3) diagnosis of IDA; (4) attending antenatal clinic in the first half of pregnancy; and (5) absence of other medical or chronic underlying disorders. The control group met the same inclusion criteria, except that the women were non-anemic and considered low-risk.

Exclusion criteria were: (1) multifetal pregnancies; (2) anemia due to causes other than iron deficiency, including thalassemia, hemolytic anemia, vitamin B12 deficiency, folic acid deficiency or sickle cell anemia, etc.; (3) pregnancies complicated by chronic illnesses that might independently affect outcomes, such as chronic kidney disease, pre-existing hypertension, diabetes, thyroid disorders, or autoimmune diseases; (4) severe pregnancy complications unrelated to anemia, including trauma, severe congenital anomalies, or chromosomal abnormalities; and (5) cases with insufficient data or uncertain outcomes.

**Research procedures** were conducted as follows: (1) The obstetric database of the Maternal–Fetal Medicine Unit was accessed to retrieve all consecutive records of pregnant women who delivered between 2003 and 2024 (a 22-year period). (2) Each record was reviewed and validated according to the inclusion and exclusion criteria. (3) Cases in the study group (iron deficiency anemia, IDA) were further examined, and the diagnosis and treatment were verified using full medical records. (4) The recruited pregnancies were categorized into two groups including ***Control group***: non-anemic, low-risk pregnant women with hemoglobin (Hb) > 11 g/dL at the first visit in the first trimester or Hb > 10.5 g/dL at the first visit in the second trimester, and ***Study group***: Pregnant women with IDA, defined as Hb < 11 g/dL at the first visit in the first trimester or Hb < 10.5 g/dL at the first visit in the second trimester, together with laboratory confirmation of IDA. (5) Baseline and outcome measures were collected as follows: Maternal baseline characteristics: maternal age (years), parity, pre-pregnancy BMI, gestational age at first visit and at delivery (weeks), socioeconomic status, ethnicity, residency, and smoking status; Laboratory parameters: Hb levels (g/dL) at three time points (first visit, early third trimester, and on admission for delivery); mean corpuscular volume (fL) for thalassemia screening at the first visit; Labor and delivery outcomes: mode of delivery, labor induction, total weight gain, estimated blood loss (mL), and maternal complications; Primary outcome: prevalence of fetal growth restriction (FGR); Secondary outcomes: preterm birth, low birth weight, preeclampsia, cesarean section rate, antepartum hemorrhage, perinatal mortality, postpartum hemorrhage, maternal blood transfusion, and fetal outcomes (birth weight, sex, gestational age at delivery, Apgar scores). (6) In our routine practice, patients with IDA were counseled separately regarding the need for therapy and follow-up and were subsequently managed in a high-risk antenatal clinic. At each follow-up visit, adherence to therapy was assessed through both direct measures, such as evaluation of Hb levels, and indirect methods, including patient questionnaires, self-reports, pill counts, prescription refill rates, and assessment of clinical response.

**Definitions** used in this study followed the ACOG guidelines [14], as follows: *(1) Maternal anemia:* Hb < 11 g/dL in the first and third trimesters or Hb < 10.5 g/dL in the second trimester. *(2) Iron deficiency anemia (IDA):* anemia with serum ferritin < 30 ng/mL, or serum ferritin 30–50 µg/L with serum iron < 50 µg/dL. *(3) Fetal growth restriction (FGR):* birth weight < 10th percentile for gestational age, based on the Thai fetal growth chart. *(4) Preterm birth:* delivery before 37 weeks and after 20 weeks of gestation. *(5) Low birth weight:* birth weight < 2500 g for deliveries after 20 weeks of gestation. *(6) Preeclampsia:* new-onset hypertension after 20 weeks of gestation with proteinuria (24 h urine protein > 300 mg or equivalent). *(7) Perinatal mortality:* intrauterine death after 20 weeks of gestation or neonatal death within 7 days of life. *(8) Postpartum hemorrhage:* estimated blood loss > 500 mL for vaginal delivery or >1000 mL for cesarean delivery. *(9) Low Apgar score:* score < 7 at 1 and 5 min.

**Statistical analysis**: The validated data were analyzed using the Statistical Package for the Social Sciences (SPSS), version 26.0 (IBM Corp., Released 2019; IBM SPSS Statistics for Windows, Version 26.0). Continuous variables are presented as mean ± standard deviation (SD) or median with interquartile range (IQR), depending on the distribution, while categorical variables are presented as frequencies and percentages. For comparisons of demographic characteristics and obstetric outcomes, categorical variables were analyzed using the chi-square test with relative risk (RR) and 95% confidence intervals (CIs). Continuous variables were compared using Student’s t test or the Mann–Whitney U test, as appropriate. Multivariate analysis was performed using binary logistic regression to identify independent risk factors for outcomes that were statistically significant in univariate analysis. Adjusted odds ratios (ORs) with 95% CIs were calculated to estimate relative risk. A *p*-value of <0.05 was considered statistically significant.

Based on a prevalence of fetal growth restriction of 7.6% among low-risk pregnancies in our hospital and an expected prevalence of approximately 10.8% in the IDA group, the sample size calculation for comparing two independent proportions indicated that approximately 688 IDA cases were required to achieve 80% power at a 95% confidence level.

## 3. Results

**Figure 1:**
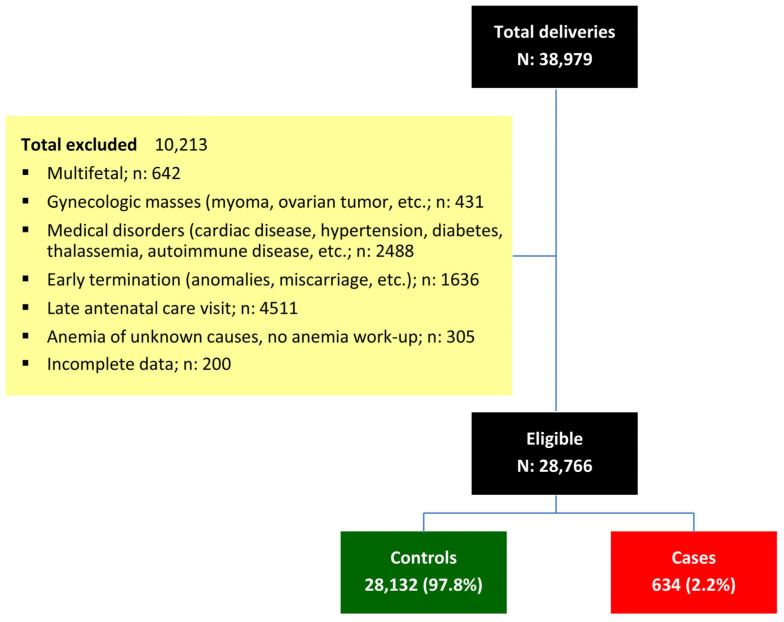
Flowchart of patient recruitment and categorization.

During the study period, a total of 38,979 pregnant women attended our antenatal care clinic and delivered at our hospital. Of these, 10,213 were excluded for various reasons, as shown in Figure 1. Ultimately, 28,766 women were included in the analysis, comprising 28,132 (97.8%) non-anemic controls and 634 (2.2%) cases with IDA.

Baseline characteristics of the patients are summarized in Table 1. Hemoglobin (Hb) levels in the IDA group were significantly lower than those in the control group at all three time points (first visit, early third trimester, and admission for delivery). The IDA group also had a significantly higher proportion of parous women (50.3% vs. 43.7%) and women of low socioeconomic status (57.4% vs. 53.1%), whereas other demographic variables were comparable between the two groups.

Pregnancy outcomes of the study and control groups are presented in Table 2. The mean gestational age and birth weight were slightly but significantly lower in the IDA group compared with the control group. Although most pregnancy outcomes were comparable between the two groups, the rates of preterm birth, fetal growth restriction, and low birth weight were significantly higher in the IDA group, with odds ratios of 1.27 (95% CI: 1.07–1.50), 1.38 (95% CI: 1.06–1.81), and 1.38 (95% CI: 1.17–1.63), respectively.

For the three pregnancy outcomes that showed significant differences (preterm birth, fetal growth restriction, and low birth weight), logistic regression analyses were performed to determine whether they were independent risk factors, as presented in Table 3. After adjusting for maternal age, pre-pregnancy BMI, parity, and socioeconomic status, the rates of preterm birth, fetal growth restriction, and low birth weight remained slightly but significantly higher in the IDA group, with odds ratios of 1.04 (95% CI: 1.01–1.07), 1.02 (95% CI: 1.00–1.04), and 1.05 (95% CI: 1.03–1.08), respectively.

## 4. Discussion

The key insight from this study is that IDA, even when treated, is associated with a modestly increased risk of pregnancy outcomes, particularly preterm birth, FGR, and low birth weight. In contrast, other adverse outcomes, most notably postpartum hemorrhage, which was of particular concern, did not differ significantly between the two groups. The observed increase in risk was minimal yet significant, with adjusted odds ratios ranging from 1.02 to 1.04. This increased risk was consistent with significantly lower gestational age and birth weight, thereby reinforcing the reliability of the findings. The adjusted odds ratios indicated that treated IDA was associated with a modestly increased risk of adverse outcomes, which was statistically significant but likely of limited clinical significance. These results may indirectly suggest that early detection and iron treatment are effective but not entirely sufficient, possibly due to suboptimal compliance among some patients.

It has been well documented worldwide that iron deficiency anemia (IDA), particularly when present in early gestation, is significantly associated with adverse neonatal outcomes, including preterm birth, fetal growth restriction (FGR), and low birth weight [9,15,16,17,18,19], as well as adverse maternal outcomes such as increased blood loss, the need for blood transfusion, and maternal infection [20,21]. Collectively, these findings underscore the critical importance of maintaining adequate iron status for optimal fetal growth and development, especially during the early stages of pregnancy. However, whether treatment of IDA can effectively prevent such adverse outcomes has not been thoroughly investigated. Therefore, we conducted this study to highlight pregnancy outcomes following the treatment of IDA. Although treatment of IDA initiated in early pregnancy was expected to equalize outcomes, women with IDA still experienced a modest but significant increase in adverse outcomes. This suggests that while treatment reduces risk, it may not fully eliminate it, likely due to inadequate correction of anemia. The results reinforce the importance of timely and effective management, and highlight practical challenges such as poor compliance with oral iron, the need for parenteral therapy in selected cases, and coexisting nutritional deficiencies, particularly in women from lower socioeconomic backgrounds.

It should be noted that the prevalence of IDA in this study was very low (2.2%), which does not reflect the true prevalence. This underestimation is likely because several IDA cases were not included, either due to the absence of laboratory work-up, since clinicians often initiated a therapeutic trial in cases with a high clinical suspicion of IDA, or because many women first attended our antenatal care clinic in the latter half of pregnancy.

Notably, Iron treatment in this study was limited to oral iron, which remains the standard approach worldwide, particularly in low-resource settings. Although intravenous iron is increasingly used in pregnancy and has shown greater improvement in hematological parameters than oral iron [22], its effect on serious adverse outcomes remains uncertain. A recent Cochrane review found little to no difference in adverse pregnancy outcomes between intravenous and oral iron and highlighted the need for large, high-quality trials to confirm these findings [13,23]. In our experience, only a few patients received parenteral iron, mainly due to intolerance to oral formulations or the need for rapid hemoglobin correction in severe anemia diagnosed in late gestation.

**Limitations and strengths**: (1) Its retrospective design restricts the ability to explore certain details and may affect data reliability. Due to this design, measures of medication adherence may not have been as rigorous as those typically applied in prospective research settings, where research physicians can closely monitor patients through telephone or e-mail follow-up to assess compliance, detect side effects, and provide timely support, particularly for those who miss scheduled visits. Nevertheless, the results of this study may more accurately reflect outcomes in real-world practice and provide valuable insights for improving future implementation strategies. (2) The sample size was relatively small to detect subtle differences, if any, particularly for rare outcomes such as postpartum hemorrhage. The strengths of this study include: (1) a large sample size, providing sufficient statistical power; and (2) a relatively homogeneous study population in terms of diagnosis and treatment protocol. In contrast to most previous studies with heterogeneous populations and limited assessment of treatment adequacy, this study excluded women initiating antenatal care in late pregnancy to ensure comparable treatment exposure. Thus, the findings more accurately reflect pregnancy outcomes in treated IDA.

**Research implications**: Several previous studies have reported associations between iron deficiency anemia (IDA) and increased risks of low birth weight, preterm delivery, perinatal mortality, instrumental delivery, postpartum hemorrhage, and maternal blood transfusion [7,9,24]. Moreover, maternal IDA has been linked to postpartum depression as well as impaired mental and psychomotor performance in offspring [25,26,27,28]. However, most studies included heterogeneous populations, encompassing both untreated IDA and treated IDA with uncertain adequacy. Therefore, further research is urgently needed—particularly studies that account for treatment effects and interventional studies at the epidemiological level.

## 5. Conclusions

IDA, even with treatment, may still slightly increase the risk of adverse pregnancy outcomes, particularly preterm birth, fetal growth restriction, and low birth weight. Although such outcomes are largely preventable, the modestly increased risk observed in this study was likely attributable to incomplete correction of anemia. Effective management requires ensuring treatment compliance, considering the use of parenteral iron when appropriate, and addressing coexisting nutritional deficiencies, particularly in vulnerable populations. These challenges underscore the need for heightened attention, more intensive monitoring, and the development of improved care strategies.

## Figures and Tables

**Table 1:** Baseline characteristics of the patients in the study and control groups.

Baseline Characteristics	Controls (n = 28,132)	Iron Deficiency Anemia (n = 634)	*p*-Value
Maternal age (year) (mean ± SD)	28.4 ± 6.8	28.6 ± 6.0	0.345
Pre-pregnancy BMI: Kg/m^2^	21.6 ± 3.5	21.3 ± 3.3	0.053
Parity (Nulliparous/parous)	15,842/12,290 (56.3%/43.7%)	315/319 (49.7%/50.3%)	0.001
Hb at first visit: gm/dL; (mean ± SD)	13.0 ± 1.6	8.9 ± 1.9	<0.001
Hb early third trimester: gm/dL; (mean ± SD)	12.1 ± 0.9	11.9 ± 1.6	<0.001
Hb before delivery: gm/dL; (mean ± SD)	13.1 ± 1.3	12.2 ± 1.3	<0.001
Residency (Others/Chiang Mai)	8111/20,021 (28.8%/71.2%)	180/454 (28.4%/71.6%)	0.809
Socioeconomic status (High/Low)	13,181/14,951 (46.9%/53.1%)	270/364 (42.6%/57.4%)	0.033
Fetal sex (Male/Female)	14,658/13,439 (52.2%/47.8%)	322/309 (51.0%/49.0%)	0.571

**Table 2:** Pregnancy outcomes of the patients in the study and control groups.

Pregnancy Outcomes	Controls (n = 28,132)	Iron Deficiency Anemia (n = 634)	*p*-Value	Relative Risk (95% CI)
Gestational age (weeks)	37.9 ± 2.4	37.6 ± 2.5	0.001	
Birth weight (g)	2965 ± 665	2864 ± 582	<0.001	
Preeclampsia	1439 (5.1%)	28 (4.4%)	0.429	0.86 (0.60–1.24)
Antepartum hemorrhage	909 (3.2%)	19 (2.9%)	0.741	0.98 (0.59–1.45)
Cesarean section	7065 (25.1%)	140 (22.1%)	0.081	0.88 (0.79–1.02)
Preterm birth	4052 (14.4%)	116 (18.3%)	<0.001	1.27 (1.07–1.50)
Fetal growth restriction	1602 (5.7%)	50 (7.9%)	<0.001	1.38 (1.06–1.81)
Low birth weight	3915 (13.9%)	122 (19.2%)	<0.001	1.38 (1.17–1.63)
Low Apgar scores at 5 min (<7)	838 (2.9%)	19 (3.0%)	0.981	1.01 (0.64–1.57)
Estimated blood loss (mL)	350 ± 267	339 ± 250	0.310	
Postpartum hemorrhage	2960 (10.5%)	58 (9.1%)	0.264	0.87 (0.68–1.11)
Fetal anomalies	539 (1.9%)	13 (2.0%)	0.807	1.07 (0.62–1.84)

**Table 3:** Logistics regression analysis to evaluate risk factors for preterm birth, fetal growth restriction and low birth weight.

	Univariable Analysis		Multivariable Analysis	
**Potential Risk Factors for Preterm Birth**	***p*** **-Value**	**Odds Ratio** **(95% CI)**	***p*-Value**	**Adjusted Odds** **Ratio (95% CI)**
Iron deficiency anemia	0.006	1.04 (1.01–1.07)	0.008	1.04 (1.01–1.07)
Maternal age	<0.001	0.99 (0.99–0.99)	0.153	1.00 (0.99–1.00)
Pre-pregnancy BMI	0.871	1.00 (0.99–1.00)	0.934	1.00 (0.99–1.00)
Parity	0.437	1.00 (0.99–1.01)	0.785	0.99 (0.98–1.01)
Socioeconomic status (Low: High)	<0.001	1.05 (1.04–1.06)	<0.001	1.05 (1.04–1.06)
**Potential Risk Factors for Fetal** **Growth Restriction**	***p*** **-Value**	**Odds Ratio** **(95% CI)**	***p*** **-Value**	**Adjusted Odds** **Ratio (95% CI)**
Iron deficiency anemia	0.019	1.02 (1.00–1.04)	0.013	1.02 (1.00–1.04)
Maternal age	0.004	0.99 (0.99–0.99)	0.047	1.00 (1.00–1.00)
Pre-pregnancy BMI	0.382	1.00 (0.99–1.00)	0.381	1.00 (0.99–1.00)
Parity	<0.001	0.97 (0.97–0.98)	<0.001	0.97 (0.96–0.98)
Socioeconomic status (Low: High)	<0.001	1.01 (1.01–1.02)	<0.001	1.02 (1.01–1.02)
**Potential Risk Factors for Low Birth Weight Newborns**	***p*** **-Value**	**Odds Ratio** **(95% CI)**	***p*** **-Value**	**Adjusted Odds** **Ratio (95% CI)**
Iron deficiency anemia	<0.001	1.05 (1.02–1.08)	<0.001	1.05 (1.03–1.08)
Maternal age	0.001	0.99 (0.99–0.99)	0.265	1.00 (1.00–1.00)
Pre-pregnancy BMI	0.598	1.00 (0.99–1.00)	0.630	1.00 (0.99–1.00)
Parity	<0.001	0.97 (0.96–0.98)	<0.001	0.97 (0.96–0.97)
Socioeconomic status (Low: High)	<0.001	1.04 (1.03–1.05)	<0.001	1.12 (1.08–1.16)

## Data Availability

The datasets analyzed during the current study are available from the corresponding author upon reasonable request.
